# Behavioral and Histopathological Impairments Caused by Topical Exposure of the Rat Brain to Mild-Impulse Laser-Induced Shock Waves: Impulse Dependency

**DOI:** 10.3389/fneur.2021.621546

**Published:** 2021-05-21

**Authors:** Motoyuki Jitsu, Katsuki Niwa, Go Suzuki, Takeyuki Obara, Yukiko Iwama, Kohsuke Hagisawa, Yukihiro Takahashi, Yoshitaro Matsushita, Satoru Takeuchi, Hiroshi Nawashiro, Shunichi Sato, Satoko Kawauchi

**Affiliations:** ^1^Military Medicine Research Unit, Japan Ground Self Defense Force, Tokyo, Japan; ^2^Department of Neurosurgery, National Defense Medical College, Tokorozawa, Japan; ^3^Division of Bioinformation and Therapeutic Systems, National Defense Medical College Research Institute, Tokorozawa, Japan

**Keywords:** diffuse axonal injury, elevated plus maze test, forced swimming test, spontaneous motor activity, blast-induced traumatic brain injury, laser-induced shock wave

## Abstract

Although an enormous number of animal studies on blast-induced traumatic brain injury (bTBI) have been conducted, there still remain many uncertain issues in its neuropathology and mechanisms. This is partially due to the complex and hence difficult experimental environment settings, e.g., to minimize the effects of blast winds (tertiary mechanism) and to separate the effects of brain exposure and torso exposure. Since a laser-induced shock wave (LISW) is free from dynamic pressure and its energy is spatially well confined, the effects of pure shock wave exposure (primary mechanism) solely on the brain can be examined by using an LISW. In this study, we applied a set of four LISWs in the impulse range of 15–71 Pa·s to the rat brain through the intact scalp and skull; the interval between each exposure was ~5 s. For the rats, we conducted locomotor activity, elevated plus maze and forced swimming tests. Axonal injury in the brain was also examined by histological analysis using Bodian silver staining. Only the rats with exposure at higher impulses of 54 and 71 Pa·s showed significantly lower spontaneous movements at 1 and 2 days post-exposure by the locomotor activity test, but after 3 days post-exposure, they had recovered. At 7 days post-exposure, however, these rats (54 and 71 Pa·s) showed significantly higher levels of anxiety-related and depression-like behaviors by the elevated plus maze test and forced swimming test, respectively. To the best of the authors' knowledge, there have been few studies in which a rat model showed both anxiety-related and depression-like behaviors caused by blast or shock wave exposure. At that time point (7 days post-exposure), histological analysis showed significant decreases in axonal density in the cingulum bundle and corpus callosum in impulse-dependent manners; axons in the cingulum bundle were found to be more affected by a shock wave. Correlation analysis showed a statistically significant correlation between the depression like-behavior and axonal density reduction in the cingulum bundle. The results demonstrated the dependence of behavior deficits and axonal injury on the shock wave impulse loaded on the brain.

## Introduction

The risk of suffering from blast-induced traumatic brain injury (bTBI) continues not only for military personnel but also for civilians due to frequent attacks using improvised explosive devices (IEDs) (1–4). Most of the patients lack any abnormality in conventional neuroimaging for diagnosis, usually computed tomography, and they have therefore been diagnosed as having mild bTBI (5–7). However, many of them have developed chronic, persistent neuropsychiatric symptoms (8–10) as well as many other symptoms such as headache (11) and sleep disorder (12), causing a serious decrease in their quality of life. Although extensive studies have been conducted, the neuropathology and mechanisms of bTBI have not been fully elucidated (13). This is partially due to the complex and hence difficult experimental environment settings. In animal studies, it is important to examine the primary mechanism (effects of a shock wave itself) since the above-mentioned bTBI-related symptoms have been observed in patients free from interactions with the secondary mechanism (effects of propelled debris and shrapnel) and the tertiary mechanism (effects of acceleration due to blast winds) (14). Thus, the effects of wind or a jet should be carefully minimized. Although bTBIs are injuries due to systemic exposure in most cases (14), the outcomes of systemic exposure are too complex to analyze. In animal studies, therefore, it is occasionally necessary to separate the effects of brain exposure from those of torso exposure to examine the effects of brain exposure alone.

We have been using laser-induced shock waves (LISWs) to investigate the mechanisms of shock wave-induced brain injury (15–17). An LISW is a pure shock wave that is free from wind or a jet. In addition, the energy of an LISW is spatially well confined and its size is controllable by changing the laser spot size, enabling site-specific shock wave application. Thus, we can exclude both the effects of the tertiary mechanism and torso exposure in animal experiments by using LISWs.

The purpose of this study was to examine behavioral and neuropathological changes caused by exposure of the brain alone to LISWs as a function of shock wave impulse in rats. Impulse is defined as the time-integrated positive pressure component of a shock wave and is known to be one of the most important parameters to determine shock wave-induced tissue damage (18). Considering neuropsychiatric symptoms that have been reported in bTBI patients (19, 20), we assessed locomotor activity, anxiety-related behavior and depression-like behavior for the rats. We also examined axonal injury in the brain by histological analysis using Bodian silver staining; diffuse axonal injury has also been reported in bTBI patients (8, 21), although it is not clear how the axonal injury is unique to bTBI. Associations between the findings obtained from behavioral tests and those obtained from pathological assessment are discussed.

## Materials and Methods

All requests for animals and procedures intended to be used in the present study were approved by the Ethics Committee of Animal Care and Experimentation, National Defense Medical College, Japan (Permission numbers: 10040, 13024, and 16036).

### Animals

Mice and rats have been most widely used for studying the pathology and mechanisms of bTBI. While the genetic background has been characterized in more detail for mice, rats have a larger forebrain and show greater sophistication and sociability than mice do. Thus, it has been suggested that rats can provide more comparability with humans in neurobehavioral studies (22, 23). Considering this factor, we chose rats as model animals for the present study. Nien-week-old male Sprague-Dawley rats were obtained from Japan SLC, Inc. (Shizuoka, Japan) and were housed in standard laboratory cages on a 12:12-h light/dark cycle with free access to food and water after arrival. Rats at 10 weeks of age weighing 300–340 g (*n* = 100) were divided into a control group without LISW exposure (*n* = 16) and five LISW groups (*n* = 84); the shock wave conditions are described in detail later.

### Generation and Characteristics of Laser-Induced Shock Waves

**Figure 1:**
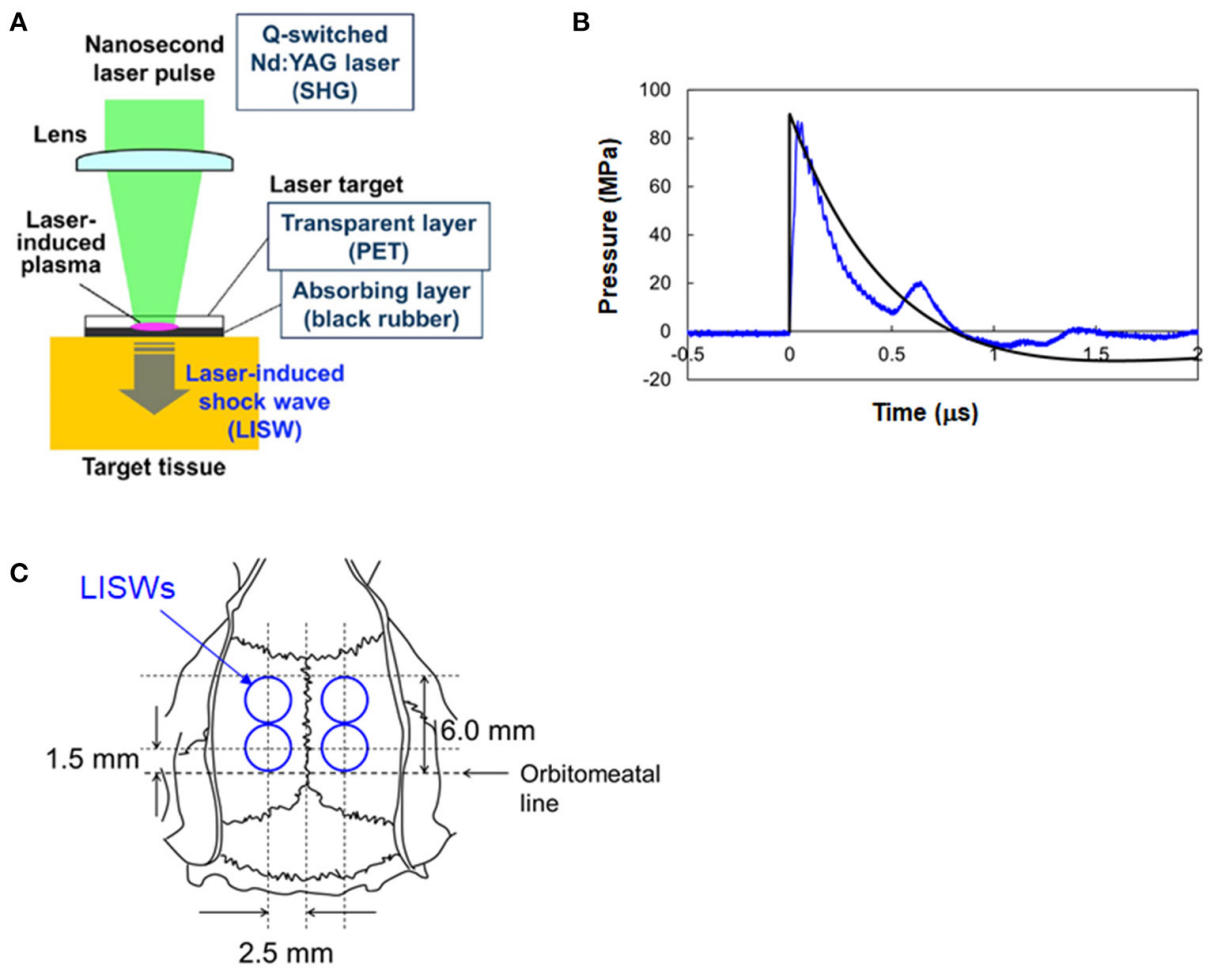
Generation and characteristics of laser-induced shock waves (LISWs) and their placement in rat brains. **(A)** Setup for generating LISW. **(B)** Typical temporal waveform of LISW (~82 MPa) with a Friedlander waveform, P = P_s_(e^(−*t*/t*)^)(1–(t/t*)) (P_s_ = 90 MPa, t* = 0.8 μs). **(C)** Positions of four LISWs transcutaneously applied to the rat brain, which are shown with respect to the skull anatomy.

A method for generating an LISW was described previously (15) (Figure 1A). Briefly, a laser target, which was a light-absorbing material (0.5-mm-thick natural black rubber disk) covered with an optically transparent material (1.0-mm-thick polyethylene terephthalate [PET] sheet) was irradiated with a high-intensity laser pulse from a Q-switched Nd:YAG laser (pulse width, 6 ns FWHM; wavelength, 532 nm; Brilliant b; Quantel, Les Ulis Cedex, France). The laser pulse was absorbed by the black rubber to induce plasma and its expansion was accompanied by the generation of a shock wave (LISW). Peak pressure and impulse of an LISW can be precisely controlled by changing the laser fluence on the target. The size of an LISW can also be controlled by changing the spot size (diameter) of the laser beam.

A problem in using LISWs for bTBI study is that the duration of positive pressure (hereafter simply called duration) of an LISW is as short as ~1 μs (Figure 1B), which is much shorter than typical IED-related shock waves (18, 24). In most of the shock tube-based bTBI studies using rodents, on the other hand, shock waves with durations of milliseconds have been used to mimic IED-related shock waves. It should be noted, however, that the interaction of real IED shock waves with a human brain and that with rodents' brains are totally different in terms of pressure gradient formed within the brain and boundary effects due to anatomical differences of the brains. The effects of shock wave duration was discussed in the Discussion section. In this study, we assumed that the impulse of the shock wave is a primary parameter to determine brain injury. Alley estimated impulses of IED-related shock waves to range from ~6.9 to ~100 Pa·s at propagation distances of 1–10 m (18). We produced 3-mm diameter LISWs with impulses ranging from 15 to 71 Pa·s by changing the laser fluence on the target in the range of 0.6~3.0 J/cm^2^. This impulse range was within the conditions that were shown to cause mild bTBI in rats by using a shock tube (<250 Pa·s) in a study by Mishra et al. (25). As described in section Neuropathology, the rats were transcardially perfused at 7 days post-exposure, and the brains were extracted. No brain hemorrhages were seen upon visual observation at any of the impulses used.

### LISW Application

Rats were anesthetized with pentobarbital sodium (i.p. 50 mg/kg) and their heads were shaved. A rat in the LISW groups was subjected to a set of four LISWs at a given impulse on the following sites to cover the whole limbic system (Figure 1C): 5 mm bilateral from a point at the intersection of the auricular line with the mid-sagittal line and then 5 mm anterior from each point. The interval between each exposure was ~5 s. For each LISW application, a laser target was placed on the scalp with forceps; ultrasound gel was applied between the bottom of the target (rubber) and the scalp for acoustic impedance matching. Rats in the LISW groups were exposed to a set of LISWs generated at laser fluences of 0.6, 1.2, 1.8, 2.4, and 3.0 J/cm^2^, the corresponding impulses (peak pressures) of LISWs being 15 Pa·s (33 MPa), 29 Pa·s (52 MPa), 43 Pa·s (66 MPa), 54 Pa·s (76 MPa), and 71 Pa·s (82 MPa), respectively. Each rat underwent either behavioral tests or pathological assessments. The number of animals in each group is stated in the Results section. Rats in the control group (sham controls) were treated in the same way except for exposure to LISWs. They received the same anesthesia and preparation as those the rats of LISW groups did. LISWs were generated at the position ~10 cm away from the rats but were not applied to them.

### Behavioral Assessments

To assess potential behavioral changes after LISW exposure, rats underwent (i) a locomotor activity test, (ii) an elevated plus-maze test, and (iii) a forced swimming test to assess spontaneous activity, anxiety-related behavior (26) and depression-like behavior (27), respectively. Experimenter blinding was not done, but all behavioral experiment data were collected by recording systems and analyzed with a dedicated software, ensuring the objectivity of our results.

Locomotor activity of rats was measured in a 12-h dark cycle using a Supermex apparatus (Muromachi Kikai Co., Ltd., Tokyo, Japan) for 5 consecutive days from 1 day post-exposure. With the apparatus, spontaneous motor activities of individual rats can be assessed by detecting heat radiation from the body with a far-infrared sensor attached to the cage (28).

At 7 days after LISW exposure, rats were subjected to an elevated plus maze test (Model EPM-04R, Muromachi Kikai Co., Ltd., Tokyo, Japan). The open arms and closed arms were both 50 cm in length and 10 cm in width; the wall height and stand height were 40 and 50 cm, respectively. After acclimatization to the experimental room for at least 1 h, each rat was placed on the center platform facing one of the two open arms and allowed to behave freely for 5 min. Behavior was recorded with a video tracking system (DVTrack Video Tracking System CompACT VAS/DV; Muromachi Kikai Co., Ltd., Tokyo, Japan), and the percentage of time spent in the open arms to the total time and total distance traveled were analyzed.

Thereafter, rats were allowed to rest for more than 1 h and were then subjected to a forced swimming test using a cylindrical tank 60 cm high and 30 cm in diameter. We based on a published protocol in which a single session was used and results for the first vigorous phase was excluded (29). Each rat was placed in 25°C water in a plastic cylinder (water depth, 35 cm; diameter, 30 cm) and allowed to behave freely for 15 min. For the last 10 min, the time spent immobile was measured and its percentage to the total time (10 min) was calculated, for which ImageJ PS1, a software package modified on the basis of the public domain ImageJ program (developed at the U.S. National Institutes of Health and available at: http://rsb.info.nih.gov/ij) (30) (O'Hara and Co., Ltd., Tokyo, Japan), was used.

### Neuropathology

At 7 days post-exposure, rats were deeply anesthetized with pentobarbital sodium (i.p. 100 mg/kg) and were transcardially perfused with normal saline followed by 4% buffered paraformaldehyde. Brains were removed after perfusion and immersed in 4% buffered paraformaldehyde overnight and then embedded in paraffin. Five-μm-thick coronal sections at 3.3 mm posterior to the bregma were used for Bodian silver staining. Axonal densities were quantified by assessing light transmittance for digital images of the brain specimens after staining (31) using ImageJ software described above. Images were converted into 8-bit gray scale images and the mean light transmittance within the regions of interest was determined (arbitrary units, ranging from 0 to 255). High values (increased light transmittance) correspond to low staining intensity, indicating decreased axonal densities and hence axonal injury.

### Statistical Analysis

Data are presented as means ± standard deviation (SD). The results of the spontaneous movement test were analyzed by two-way repeated measures ANOVA with the Bonferroni *post-hoc* test. The results of the elevated plus maze test and forced swimming test and the light transmittance of brain specimens were analyzed by one-way factorial ANOVA with Dunnett's *post-hoc* test. The correlation between the result of the light transmittance of brain specimens and the results of the behavioral tests, i.e., the elevated plus maze test and the forced swimming test, were analyzed by Pearson's correlation coefficients. These statistical analyses were performed using GraphPad Prism software (Prism 6 for Windows, GraphPad software, Inc., La Jolla, CA, USA). A difference was considered statistically significant when *p* ≤ 0.05. To assess the validity of the sample sizes used in behavioral tests, *post-hoc* power analysis was performed using a program G^*^Power 3 (32).

## Results

### Spontaneous Locomotor Activity

**Figure 2:**
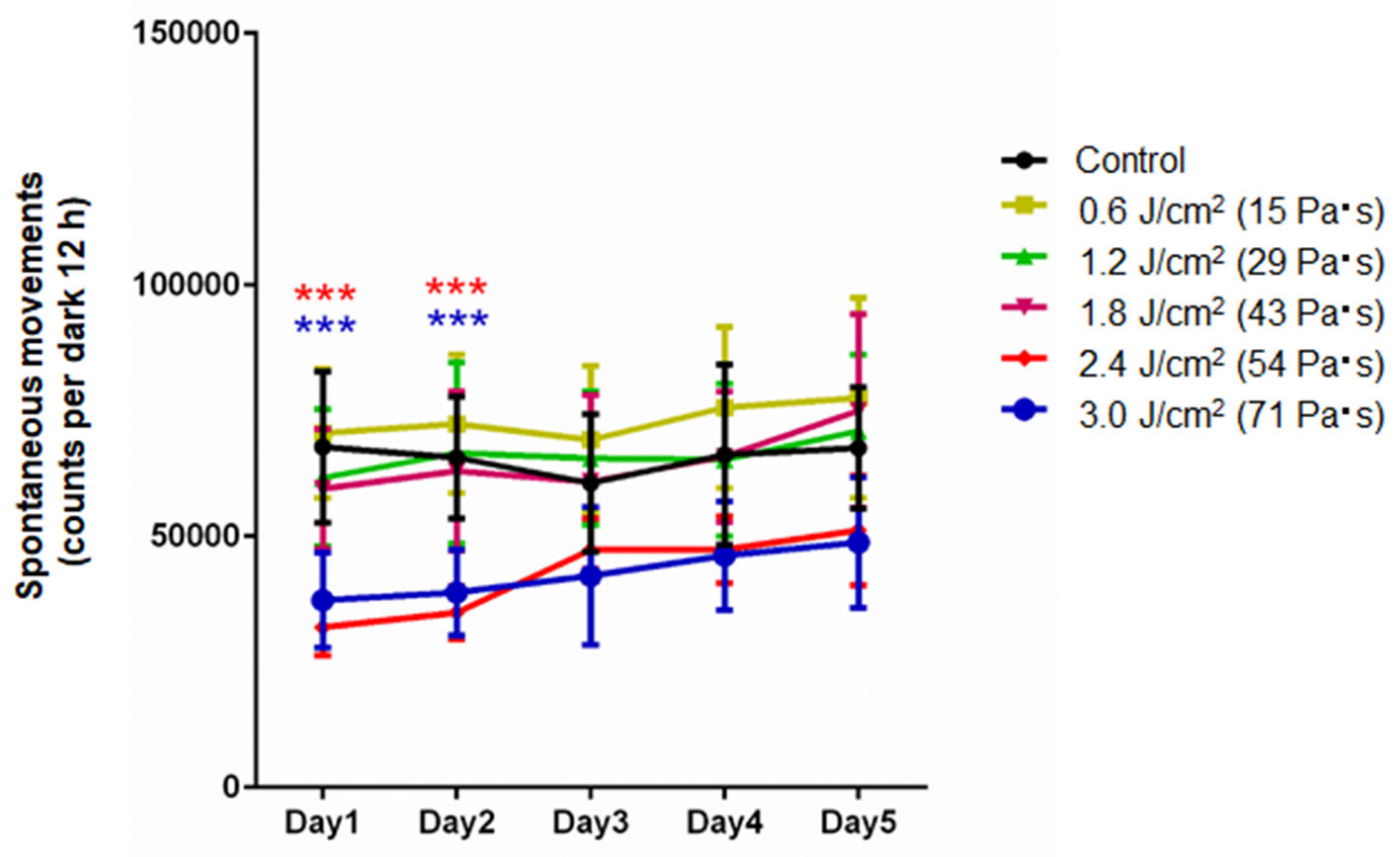
Spontaneous motor activities in the locomotor activity test as a function of post-exposure days (Day 1–5) for rats whose brains were exposed to LISWs with different impulses (*n* = 10 for 0 [control], 15, 29, and 43 Pa·s; *n* = 5 for 54 and 71 Pa·s). The data are presented as means ± SD. Asterisks (***p ≤ 0.001) indicate statistically significant differences (two-way repeated measures ANOVA with Bonferroni *post-hoc* test, vs. control).

Figure 2 shows spontaneous motor activities during 12-h dark cycles at 1–5 days post-exposure for rats in the control group and LISW groups with exposure at five different impulses (*n* = 10 for control [0 Pa·s], 15, 29, and 43 Pa·s; *n* = 5 for 54 and 71 Pa·s). The values (mean and SD) and results of the statistical analysis are summarized in Tables 1, 2. The two-way repeated measures ANOVA showed that shock wave impulses and days post-exposure had significant effects on locomotor activities; significant interaction effects were also found (*p* = 0.0042). Bonferroni *post-hoc* test showed that spontaneous motor activities were significantly decreased at 1 and 2 days post-exposure at the higher impulses of 54 and 71 Pa·s compared to those of the control group. After 3 days post-exposure, however, there were no significant differences in spontaneous motor activities between any of the LISW groups and the control group, indicating recovery of locomotor activity for LISW groups with exposure at the higher impulses.

**Table 1:** Summary of the two-way repeated measures ANOVA for the results of locomotor activity test.

**Source of variation**	***F*-value**	***P*-value**
Days post-exposure	16.88	<0.0001
Shock wave impulse	6.261	0.0002
Interaction	2.164	0.0042

**Table 2:** Summary of the results of locomotor activity test and statistical analysis with Bonferroni *post-hoc* test.

	**Mean**	**SD**	**95% confidence (vs. sham control)**	**Statistical significance**
**Day 1**				
Sham control	67,747	15,046	–	–
15 Pa·s	70,449	12,883	−17,388 to 22,793	^ns^
29 Pa·s	61,558	13,650	−26,280 to 13,901	^ns^
43 Pa·s	59,407	11,971	−28,430 to 11,751	^ns^
54 Pa·s	31,886	5,575	−60,466 to −11,255	^***^
71 Pa·s	37,221	9,444	−55,131 to −5,920	^**^
**Day 2**				
Sham control	65,633	12,132	–	–
15 Pa·s	72,327	13,805	−13,397 to 26,784	^ns^
29 Pa·s	66,558	18,015	−19,165 to 21,016	^ns^
43 Pa·s	62,928	15,784	−22,795 to 17,386	^ns^
54 Pa·s	34,749	5,280	−55,490 to −6,278	^**^
71 Pa·s	38,817	8,594	−51,421 to −2,210	^*^
**Day 3**				
Sham control	60,529	13,702	–	–
15 Pa·s	69,175	14,739	−11,445 to 28,737	^ns^
29 Pa·s	65,482	13,273	−15,137 to 25,044	^ns^
43 Pa·s	60,842	17,310	−19,778 to 20,403	^ns^
54 Pa·s	47,272	6,307	−37,863 to 11,349	^ns^
71 Pa·s	42,115	13,728	−43,020 to 6,192	^ns^
**Day 4**				
Sham control	66,194	17,945	–	–
15 Pa·s	75,545	16,046	−10,739 to 29,442	^ns^
29 Pa·s	65,177	15,173	−21,107 to 19,074	^ns^
43 Pa·s	65,855	13,042	−20,430 to 19,752	^ns^
54 Pa·s	47,297	6,687	−43,503 to 5,709	^ns^
71 Pa·s	46,098	10,823	−44,701 to 4,511	^ns^
**Day 5**				
Sham control	67,515	12,108	–	–
15 Pa·s	77,441	19,944	−10,165 to 30,016	^ns^
29 Pa·s	70,876	15,268	−16,730 to 23,451	^ns^
43 Pa·s	74,953	19,293	−12,653 to 27,528	^ns^
54 Pa·s	51,128	10,854	−40,993 to 8,218	^ns^
71 Pa·s	48,758	12,978	−43,363 to 5,849	^ns^

ns *P > 0.05*,

* *P ≤ 0.05*,

** *P ≤ 0.01*,

*** *P ≤ 0.001*.

### Anxiety-Related Behavior (Elevated Plus Maze Test)

**Figure 3:**
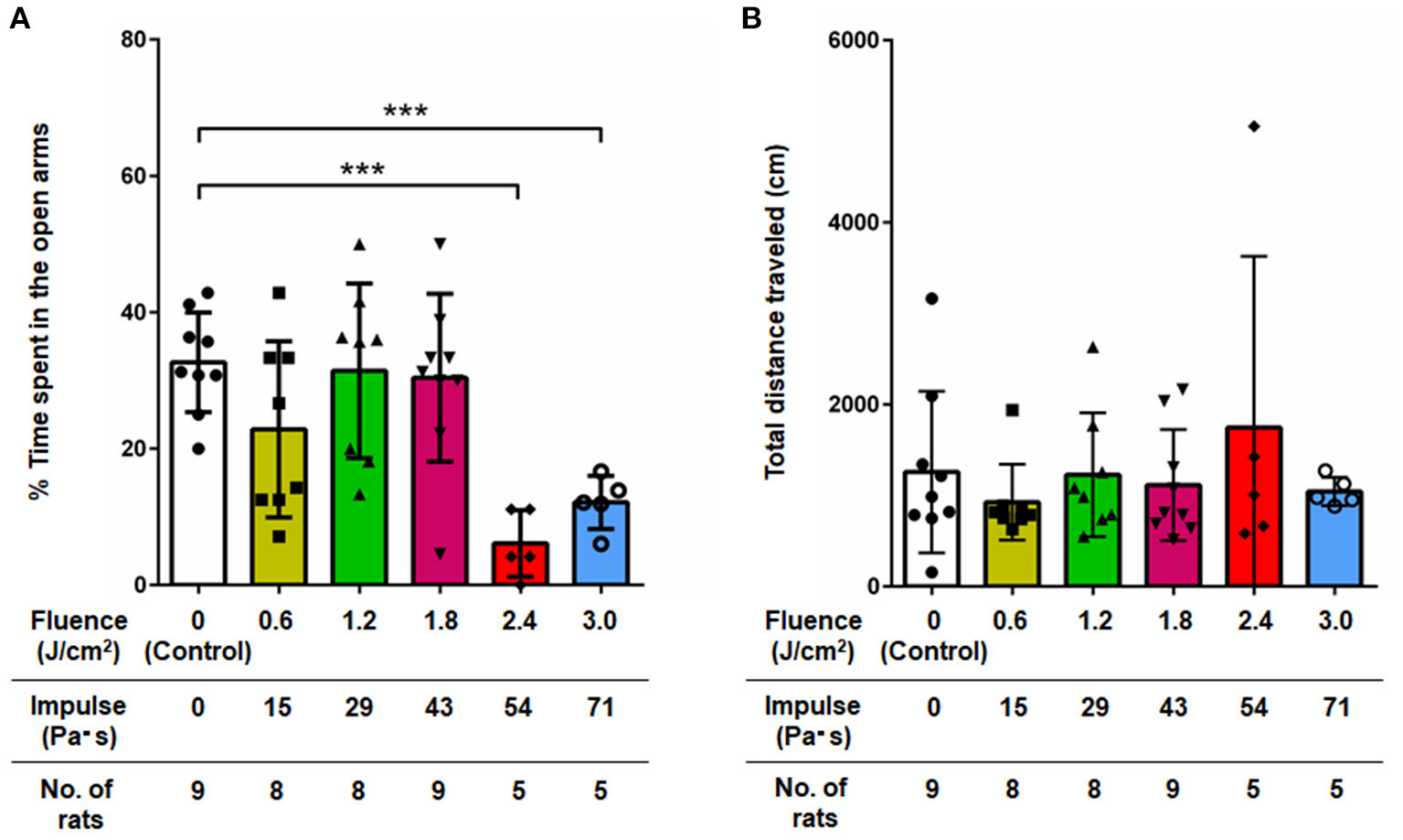
**(A)** Percentage of time spent in open arms and **(B)** total distance traveled in the elevated plus maze test at 7 days post-exposure as a function of impulses of LISWs that were applied to the rat brains (mean ± SD). Rats that fell from the arms during the test were excluded; the numbers of those rats excluded were 1, 2, 2, 1, 0, and 0 in the sham control group, 15, 29, 43, 54, and 71 Pa·s group, respectively. Asterisks (***p ≤ 0.001) indicate statistically significant differences (one-way factorial ANOVA with Dunnett's *post-hoc* test, vs. control).

Figure 3 shows the results of the elevated plus maze test for all groups of rats at 7 days post-exposure (*n* = 10 for control [0 Pa·s], 15, 29 and 43 Pa·s; *n* = 5 for 54 and 71 Pa·s), from which data for rats that fell from the arms were excluded (33). One-way factorial ANOVA showed a significant difference in the percentages of time spent in open arms (ANOVA *F*-value = 6.968; *p* = 0.0001) but not in the total distances traveled among the groups (ANOVA *F*-value = 0.6305; *p* = 0.6775). The *post-hoc* test results are summarized in Table 3. For rats in the control group, the percentage of time spent in the open arms was 32.7 ± 7.3% (*n* = 9). The percentages of time spent in the open arms for rats in the LISW groups with exposure at 54 and 71 Pa·s were significantly lower [6.1 ± 4.9% (*n* = 5) and 12.1 ± 3.9% (*n* = 5), respectively], indicating a significant increase in anxiety-related behavior for these groups of rats. Rats in the LISW groups with exposure at the lower impulses (15, 29, and 43 Pa·s) showed no significant decrease in the time spent in the open arms. On the other hand, no significance differences were found in the total distances traveled by all rat groups in elevated plus maze test, suggesting no significant changes in their locomotor activities.

**Table 3:** Summary of the results of elevated plus maze test and statistical analysis with Dunnett's *post-hoc* test.

	**Mean**	**SD**	**95% confidence (vs. sham control)**	**Statistical significance**
**%Time spent in the open arms**				
Sham control	32.7	7.3	–	–
15 Pa·s	22.8	12.9	−3.436 to 23.09	^ns^
29 Pa·s	31.4	12.8	−12.02 to 14.51	^ns^
43 Pa·s	30.4	12.3	−10.61 to 15.13	^ns^
54 Pa·s	6.1	4.9	11.32 to 41.77	^***^
71 Pa·s	12.1	3.9	5.301 to 35.75	^**^
**Total distance traveled**				
Sham control	1,257	888	–	–
15 Pa·s	926	416	−762.5 to 1,425	^ns^
29 Pa·s	1,227	680	−1,064 to 1,124	^ns^
43 Pa·s	1,114	609	−918.2 to 1,204	^ns^
54 Pa·s	1,746	1,879	−1,745 to 766.2	^ns^
71 Pa·s	1,043	156	−1,041 to 1,470	^ns^

ns *P > 0.05*,

** *P ≤ 0.01*,

*** *P ≤ 0.001*.

### Depression-Like Behavior (Forced Swimming Test)

**Figure 4:**
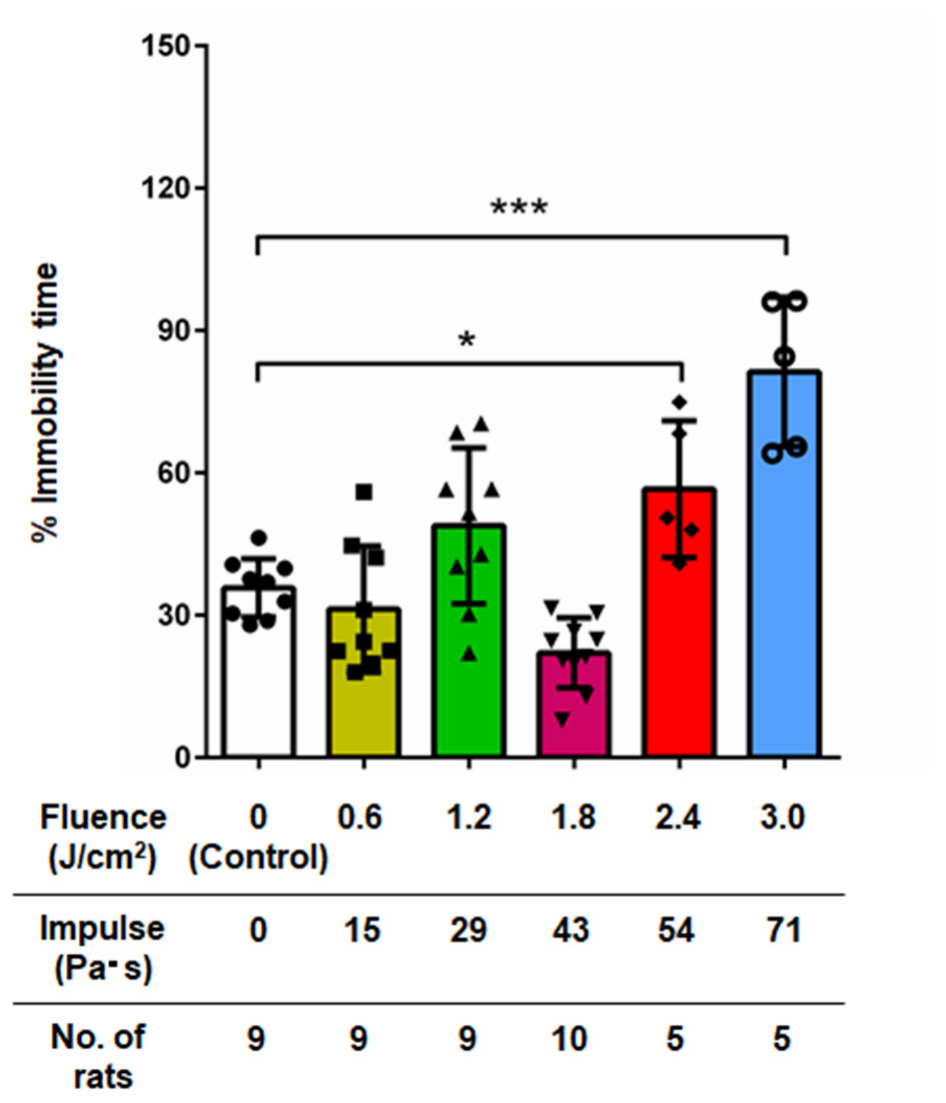
Percentage of immobility time in the forced swimming test at 7 days post-exposure as a function of impulses of LISWs that were applied to the rat brains (mean ± SD). Rats that were rescued from drowning during the test were excluded; the numbers of those rats excluded were 1, 1, 1, 0, 0, and 0 in the sham control group, 15, 29, 43, 54, and 71 Pa·s group, respectively. Asterisks (*p < 0.001, ***p ≤ 0.001) indicate statistically significant differences (one-way factorial ANOVA with Dunnett's *post-hoc* test, vs. control).

Figure 4 shows the results of the forced swimming test for all groups of rats at 7 days post- exposure (*n* = 10 for control [0 Pa·s], 15, 29 and 43 Pa·s; *n* = 5 for 54 and 71 Pa·s), from which data for rats rescued from drowning during the test were excluded (34). No rats were excluded due to visually recognized health problems. One-way factorial ANOVA showed a significant difference in the percentages of immobility time among the groups (ANOVA *F*-value = 19.3; *p* < 0.0001). The results of the *post-hoc* tests are summarized in Table 4. For rats in the control group, the percentage of immobility time was 35.8 ± 6.2% (*n* = 9). The percentages of immobility time for rats in the LISW groups with exposure at 51 and 71 Pa·s were 56.6 ± 14.4% (*n* = 5) and 81.3 ± 15.7% (*n* = 5), respectively, which were significantly greater than that for the control group. These results indicate significantly higher levels of depression-like behavior in these groups of rats. Rats in the LISW groups with exposure at the lower impulses (15, 29, and 43 Pa·s) showed no significant decrease in immobility time.

**Table 4:** Summary of the results of forced swimming test and statistical analysis with Dunnett's *post-hoc* test.

	**Mean**	**SD**	**95% confidence (vs. sham control)**	**Statistical significance**
**%Immobility time**				
Sham control	35.8	6.2	–	–
15 Pa·s	31.3	13.3	−10.66 to 19.62	^ns^
29 Pa·s	48.9	16.4	−28.24 to 2.048	^ns^
43 Pa·s	22.1	7.4	−1.093 to 28.42	^ns^
54 Pa·s	56.6	14.4	−38.73 to −2.898	^*^
71 Pa·s	81.3	15.8	−63.45 to −27.62	^****^

ns *P > 0.05*,

* *P ≤ 0.05*,

**** *P ≤ 0.0001*.

### Histopathological Assessments

**Figure 5:**
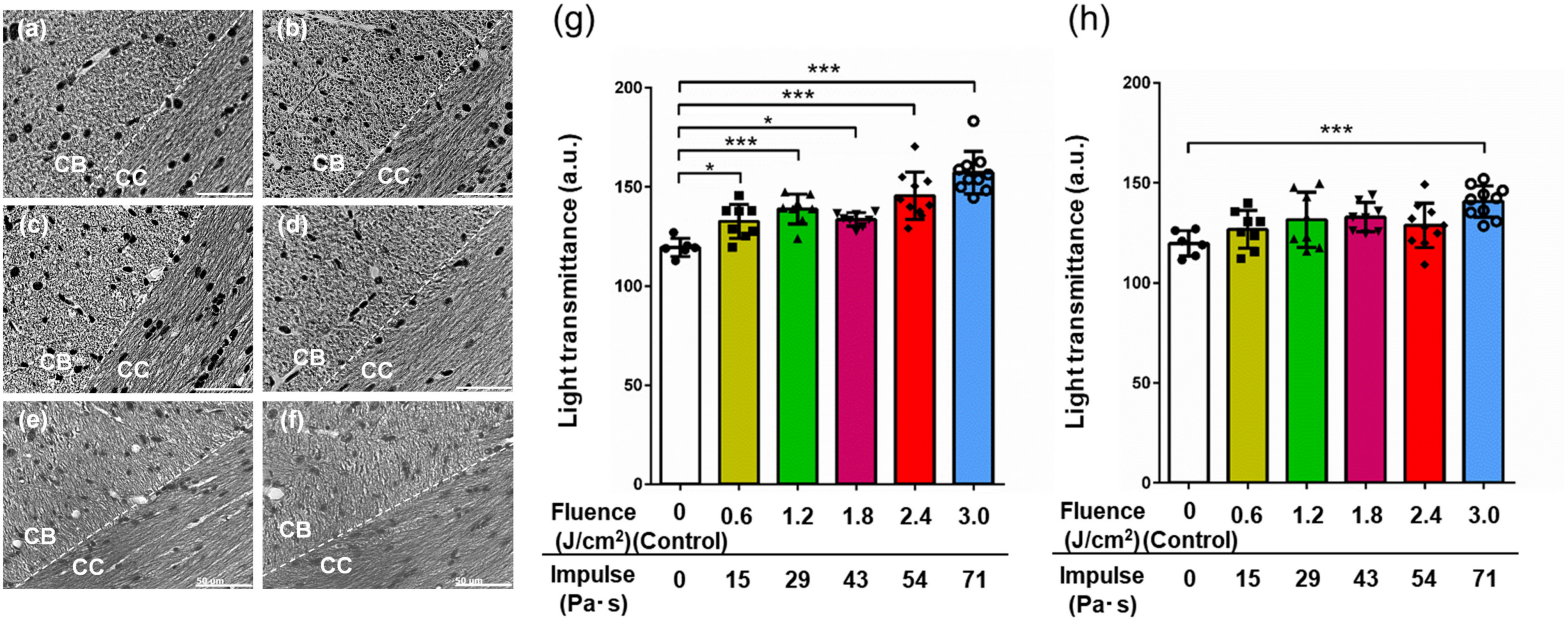
Representative images of Bodian silver-stained sections of the boundary region between the CB and the CC in brains exposed to LISWs with different impulses and their light transmittance 7 days post-exposure. **(a)** Control (0 Pa·s). **(b)** 15 Pa·s. **(c)** 29 Pa·s. **(d)** 43 Pa·s. **(e)** 54 Pa·s. **(f)** 71 Pa·s. Dashed lines indicate the boundary of the CB and CC. Scale bar indicates 50 μm. Light transmittance of Bodian silver-stained sections of the regions of **(g)** the CB and **(h)** the CC in rat brains exposed to LISWs as a function of impulse (*n* = 6 for control [0 Pa·s]; *n* = 8 for 15, 29, and 43 Pa·s; *n* = 10 for 54 and 71 Pa·s). Higher light transmittance indicates lower axonal density. The data are presented as means ± SD. Asterisks (*p ≤ 0.05, ***p ≤ 0.001) indicate statistically significant differences (one-way factorial ANOVA with Dunnett's *post-hoc* test, vs. control).

Figures 5a–f shows representative images of Bodian-stained brain sections at a boundary region of the cingulum bundle (CB) and the corpus callosum (CC) in the brains of all groups of rats. The whole image region of the control brain section is relatively dark (Figure 5a). For the sections of brains of rats in the LISW groups, on the other hand, the CB regions are lighter than the CC regions, indicating increased light transmittance and hence decreased axonal density in the CB regions (Figures 5b–f). For all of the images, a unidirectional fibrous structure is seen in the CC regions, while there are no such microstructures in the CB regions, indicating different fiber orientations in the CB and CC.

To evaluate axonal densities in the CB and CC regions in each image, light transmittance was quantified and the values are shown in Figures 5g,h, respectively (*n* = 6 for control [0 Pa·s]; *n* = 8 for 15, 29, and 43 Pa·s; *n* = 10 for 54 and 71 Pa·s). One-way factorial ANOVA showed a significant difference in light transmittances in CB (ANOVA *F*-value = 16.98; *p* < 0.0001) and CC (ANOVA *F*-value = 4.033; *p* = 0.0042). The results of the *post-hoc* test are summarized in Table 5. For the CB region, there is a tendency for increase in light transmittance, and hence axonal density decreases with increase in the impulse of LISWs. Light transmittances for all LISW groups were significantly higher than that for the control group (Figure 5g). At the highest impulse (71 Pa·s), the light transmittance was reduced by ~30% compared with the value for the control group. For the CC region, on the other hand, the extent of increased light transmittance (i.e., decreased axonal density) was smaller than that for the CB region; light transmittance was significantly higher only at the highest impulse (71 Pa·s) than that for the control (Figure 5h).

**Table 5:** Summary of the results of light transmittance of Bodian-stained brain sections and statistical analysis with Dunnett's *post-hoc* test.

	**Mean**	**SD**	**95% confidence (vs. sham control)**	**Statistical significance**
**Cingulum bundle (CB)**			–	
Sham control	119.5	4.6	–	–
15 Pa·s	132.6	8.6	−25.52 to −0.7135	^*^
29 Pa·s	138.8	7.5	−31.71 to −6.901	^***^
43 Pa·s	133.5	3.4	−26.46 to −1.652	^*^
54 Pa·s	145.5	11.9	−37.92 to −14.19	^****^
71 Pa·s	157.2	10.7	−49.61 to −25.88	^****^
**Corpus callosum (CC)**				
Sham control	119.7	6.4	–	–
15 Pa·s	126.7	9.5	−20.80 to 6.732	^ns^
29 Pa·s	131.5	13.8	−25.61 to 1.921	^ns^
43 Pa·s	132.9	7.3	−26.92 to 0.6145	^ns^
54 Pa·s	128.7	11.1	−22.16 to 4.168	^ns^
71 Pa·s	140.7	7.8	−34.12 to −7.790	^***^

ns *P > 0.05*,

* *P ≤ 0.05*,

*** *P ≤ 0.001*,

**** *P ≤ 0.0001*.

### Correlations Between Changes in Axonal Density and Behavioral Outcomes

**Figure 6:**
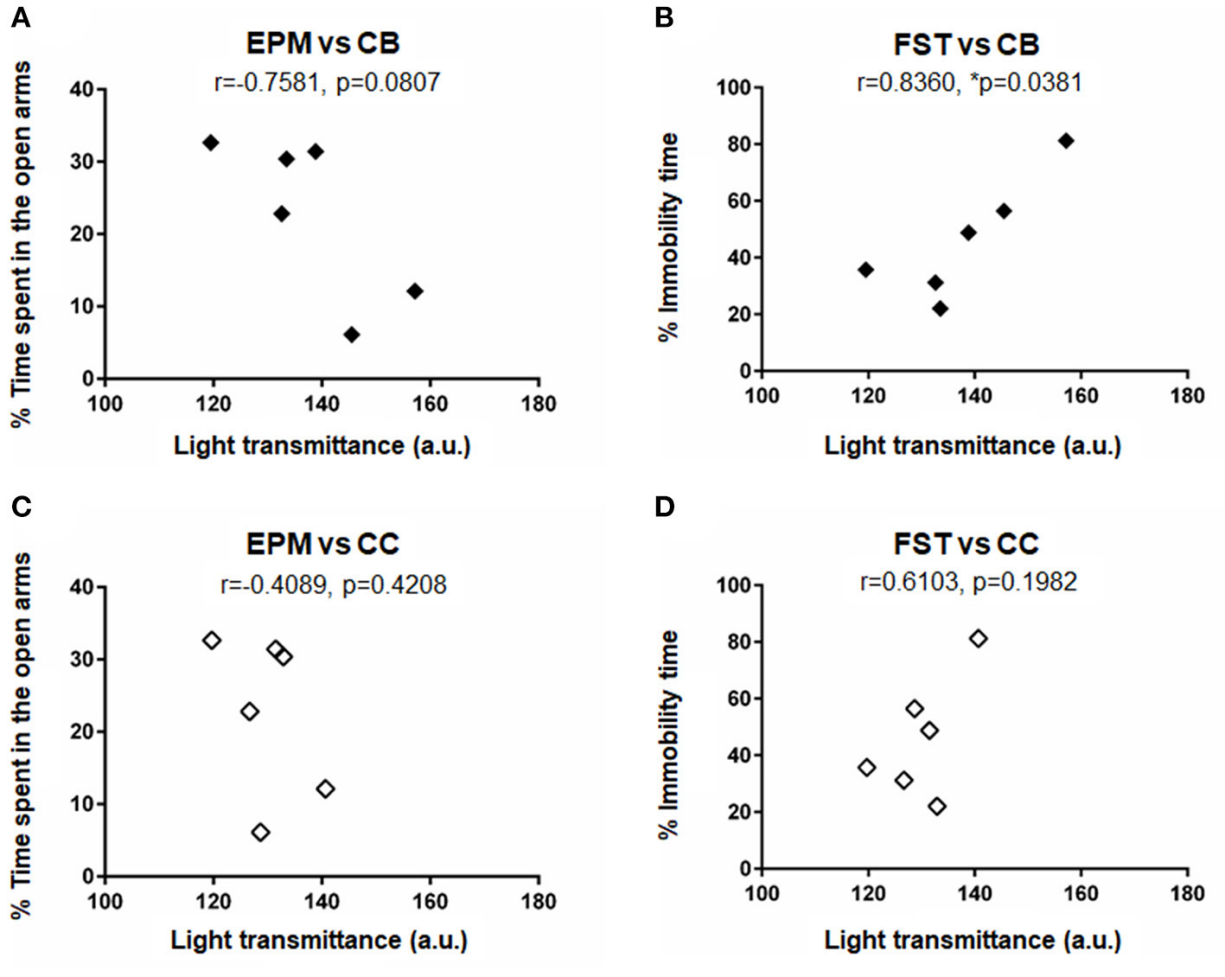
Results of correlation analysis for the data of behavioral outcomes and axonal densities: Correlations **(A)** between the light transmittance (LT) in CB and the time spent in open arms in the elevated plus maze test (EPMT), **(B)** between the LT in CB and the immobility time in the forced swimming test (FST), **(C)** between the LT in CC and the time spent in open arms in EPMT, and **(D)** between the LT in CC and the immobility time in FST. Mean data shown in Tables 2–4 were plotted for the analysis. Correlation coefficient *r* and *p*-values are shown in the figure. An asterisk (*p ≤ 0.05) indicate a statistically significant difference.

To investigate if the changes in axonal density in the CB and the CC were correlated with the behavioral outcomes, correlation analyses were performed for the data of mean light transmittance (LT) in CB and in CC, mean percentage of time spent in the open arms in the elevated plus maze test, and mean percentage of immobility time in the forced swimming test. Figure 6 shows relations between the LT in CB and the time spent in open arms (Figure 6A, *r* = −0.7581, *p* = 0.0807), between the LT in CB and the immobility time (Figure 6B, *r* = 0.8360, *p* = 0.0381), between the LT in CC and the time spent in open arms (Figure 6C, *r* = −0.4089, *p* = 0.4208) and between the LT in CC and the immobility time (Figure 6D, *r* = 0.6103, *p* = 0.1982). There was a statistically significant correlation only between the LT in CB and the immobility time in the forced swimming test, suggesting that a decreased axonal density was significantly correlated with an increased depression-like behavior.

### Verification of Sample Sizes

Since the numbers of rats in LISW groups with 51 and 71 Pa·s exposure were smaller than that in the control group, *post-hoc* power analyses were performed to verify the sample sizes. The results are shown in Table 6. In all analyses, the power was >0.8, indicating that the sample sizes in this study were acceptable.

**Table 6:** Results of *post hoc* power analysis for behavioral tests.

	**Statistical test**	**Test family**	**Effect size**	**Power**
**Locomotor activity test**				
Spontaneous motor activities	Two-way repeated measures ANOVA	*F*-tests	0.8433757	0.9904001
**Elevated plus maze test**				
%Time spent in the open arms	One-way factorial ANOVA	*F*-tests	0.716237	0.9471567
**Forced swimming test**				
%Immobility time	One-way factorial ANOVA	*F*-tests	1.072548	0.9999605

## Discussion

In this study, behavioral and neuropathological changes were evaluated for rats whose brains were topically exposed to LISWs to examine the mechanisms of shock wave-related TBI. Due to the nature of LISWs, the results are free from the effects of acceleration (tertiary mechanism) and torso exposure, purely reflecting the effect of the primary mechanism on the brain. Our rat model showed significantly lower locomotor activity in the acute phase (1 and 2 days post-exposure) at the higher impulse conditions (54 and 71 Pa·s), indicating loss of basic physical function under the unconstrained conditions. However, their locomotor function was recovered after 3 days post-exposure, and the elevated maze and forced swim tests, which were conducted at 7 days post-exposure, are therefore considered not to be affected by change in the basic physical functions. Importantly, the rats exposed to LISWs at the higher impulse conditions showed significantly higher levels of anxiety-related and depression-like behaviors at 7 days post-exposure. These behavioral changes were shown to be correlated with decreased axonal densities in CB and CC.

Some studies reported the results of anxiety tests using an elevated plus maze or elevated zero maze test for rats exposed to blast overpressures (35–39). Kovesdi et al. (35) reported that the time spent in open arms was not significantly decreased in rats at 9 days post-exposure, while it was significantly reduced (anxiety increased) in rats after 46 days in experiments using a shock tube (~20 psi [~138 kPa], single exposure). On the other hand, the experiments of Budde et al. that used a blast tube (100 and 450 kPa, single exposure) showed no significant decreases in open arm time either in the acute phase (1–4 days post-injury) or chronic phase (28–31 days post-injury) (36). Elder et al. (37) found that the rats exposed to a single 74.5-kPa blast per day for three consecutive days exhibited signs of anxiety at 40 days after exposures; rats with exposures moved less and spent significantly more time in the closed arms than in the open arms when compared with controls. Perez-Garcia et al. (38) showed for the same rat model that the blast-exposed rats spent more time in the closed arms even at 29–30 weeks after exposure. Furthermore, Blaze et al. (39) observed an anxiety-like phenotype for the same rat model at 1–1.5 months post-injury, but it was diminished by 12–13 months post-injury. The present results on elevated plus maze test can be featured by the fact that the increased rat anxiety appeared in the acute phase (7 days post-exposure). Unexpectedly, the results of the forced swimming test were limitedly reported for the rodents exposed to a blast. Kawa et al. (40) applied a blast (550 kPa, single exposure) to rats but did not observe an increase in rat immobility time either on day 1, 14, or 35. To the best of the authors' knowledge, there have been few reports on rat bTBI models that showed both anxiety-related and depression-like behaviors. A prospective, longitudinal clinical study on active-duty US military personnel who were diagnosed with mild bTBI showed that, in addition to PTSD-related symptoms, depression and anxiety were the most prominent psychiatric symptoms (20). Therefore, it can be said that anxiety-related and depression-like behaviors should be involved in important behavioral traits of bTBI animal models. Our rat model may be valuable for shock wave-related behavioral changes. The features of our model might be associated with the temporal pressure characteristic of LISWs, which was discussed below.

Goldstein et al. (41) reported that the mice exposed to a blast did not show deficits in learning and memory in the absence of rotational and/or acceleration-deceleration forces. As described above, our model was free from the effects of acceleration (tertiary mechanism) but showed behavioral alterations under high impulse conditions. This might be associated with the difference in shock wave durations: ~5 ms (shock tube) vs. ~1 μs (LISW). Although the animal species were different in the two studies, the pressure gradient for a ~1 μs-duration LISW was much steeper than that for a ~5 ms-duration shock wave, causing a greater local compression-tensile force and/or shear force in the brains.

The CB region, which was found to be affected by shock wave exposure, is the largest commissural white matter bundle in the brain and is involved in the neural circuit known as Papez circuit that controls emotional expression (42). The Papez circuit begins and ends with the hippocampus through the neural pathways via mammillary bodies, anterior thalamic nucleus and cingulum (42). Thus, axonal injury in the CB region can affect not only emotional expression but also memory and cognitive functions in individuals. Anatomically, the circuit exists in the limbic system, which should interact with the incident shock/pressure wave in the present exposure scheme. Accordingly, the observed depression-like behavior in the rats was shown to be significantly correlated with the axonal density reduction found in the CB region (Figure 6B). However, it should be noted that the current analysis is limited by the small sample size. Other structures, such as the frontal cortex and amygdala, are also involved in emotionality (43). Thus, further study is needed to fully understand the mechanisms of the observed behavioral changes.

It is interesting that the loss of axonal density was much more evident in the CB region than in the CC region (Figures 5g,h), although both the CB and CC form white matter bundles and they make contact with each other (the CB being located just above the CC). This may be associated with their axonal orientations perpendicular to each other; the axonal orientation of the CB is sagittal, while that of the CC is transverse (44). Thus, there would be a certain discontinuity of tissue mechanical properties at the CB-CC boundary, resulting in an anisotropic compliance and/or an acoustic impedance mismatching. Since LISWs were applied to the parietal region in the present study, an incident shock/pressure wave propagates from the top of the brain. This propagating pressure wave can be disturbed and reflected at the boundary, possibly generating a shear force or cavitation (2, 45). Such events might be associated with vulnerability of neuronal axons in the CB. An LISW (3 mm in diameter) propagating through the water was visualized by shadowgraphing. The diameter of the high-pressure wavefront was ~4 mm at a depth of 6 mm, indicating the relatively high directionality of LISW propagation (46). Thus, LISW application to other sites of the brain would be useful to examine the abovementioned hypothesis.

In our experiments, locomotor deficits were observed in the rats of LISW groups with exposure at 54 and 71 Pa·s only at 1 and 2 days post-exposure, while anxiety and depression-like behaviors were observed from the behavior tests conducted at 7 days post-exposure. We previously found that LISW application directly caused the occurrence of spreading depolarization, which was followed by a long-lasting oligemia/hypoxemia in the cortex (15). Since the motor cortex was involved in the region with such hemodynamic abnormality, it could affect the locomotion of rats in super-acute phase. An increase in the intracranial pressure was also observed due to the blood brain barrier disruption in this phase, which might also alter the locomotor function of rats.

There were limitations in this study. First, the observation time points were limited, being only in the acute phase. Traumatic neuropathology generally proceeds in a cascading manner, and the clinical study referred to above showed that the symptoms in the 5-year evaluation were worse than those in the 1-year evaluation (20). Thus, it is important to reveal time-dependent changes in neurobehaviors and neuropathology. Second, for neuropathology, we focused only on axonal injury, although we previously reported the increased oxidative stress in the cortex, hippocampus and cerebellum in the same model at 7 days post-exposure (17). Although the clinical neuropathology for bTBI is not fully understood, glial scar formation (47) and aberrant protein accumulation (41) have been reported on the basis of results of studies using postmortem brains of bTBI patients in addition to axonal injury. It is not clear how our neuropathological findings (axonal injury) are associated with those outcomes. Thus, comprehensive analysis of the neuropathology is needed for the rat brain in the chronic phase.

Another important issue to be addressed is the appropriate temporal characteristics of the shock wave to be applied to rodents' brains. In most of the bTBI studies using a blast tube or shock tube with rodents, shock waves with durations of milliseconds have been used to replicate actual IED-relevant shock waves (24). The positive pressure duration of a typical IED explosion-related shock wave ranges from 250 μs to several milliseconds (24). However, the interaction of the human brain with such IED-related shock waves, especially the brain to skull boundary effects, would not be reproduced in the brains of small animals when using similar shock wave duration. The size of the rat brain is roughly one-tenth of that of the human brain (in one-dimensional scale). On the basis of the scaling law, we think that the appropriate shock wave duration to be applied to the rat brain would be one-tenth of the ones described above, ranging from 25 μs to several hundred microseconds. The present LISW (~1-μs duration) was much shorter than this range, but it would still be valid to examine the brain injury associated with underwater blast-related shock waves, of which durations were reported to range from 10 to 200 μs (24). Recently, we have succeeded in controlling the duration of LISWs in a certain range. Thus, we plan to investigate the effects of different shock wave durations on the neuropathological and behavioral changes for the rats, enabling a further understanding of the results of the present study.

## Conclusions

We applied a set of four LISWs to rat brains in the impulse range of 15–71 Pa·s and examined neurobehavioral and neuropathological changes in the rats. At 7 days post-exposure, we observed significant anxiety-related and depression-like behaviors in the rats in impulse-dependent manners. Decreased axonal density was observed in both the CB and CC regions, but axons in the CB region were found to be more affected by shock wave exposure. The depression-like behavior was found to be significantly correlated with the axonal density reduction in CB. The rats characterized in this study may be used as a new model to study blast-related TBI.

## Data Availability Statement

The original contributions presented in the study are included in the article/supplementary material, further inquiries can be directed to the corresponding author/s.

## Ethics Statement

The animal study was reviewed and approved by Ethics Committee of Animal Care and Experimentation, National Defense Medical College, Japan.

## Author Contributions

MJ, KN, GS, TO, KH, YT, YM, and SS contributed to the conception, design, and administrative support of the study. MJ, KN, GS, TO, and YI performed the research and analyzed the data. MJ, ST, SS, and SK wrote the manuscript. All authors discussed and interpreted the results. All authors reviewed and approved the final manuscript.

## Conflict of Interest

The authors declare that the research was conducted in the absence of any commercial or financial relationships that could be construed as a potential conflict of interest.
